# Primary sclerosing cholangitis – The arteriosclerosis of the bile duct?

**DOI:** 10.1186/1476-511X-6-3

**Published:** 2007-01-25

**Authors:** Peter Fickert, Tarek Moustafa, Michael Trauner

**Affiliations:** 1 Laboratory of Experimental and Molecular Hepatology, Division of Gastroenterology and Hepatology, Department of Internal Medicine, Medical University of Graz, Austria

## Abstract

Primary sclerosing cholangitis (PSC) is a chronic inflammatory disease of unknown aetiology affecting the large bile ducts and characterized by periductal fibrosis and stricture formation, which ultimately result in biliary cirrhosis and liver failure. Arteriosclerosis involves the accumulation of altered lipids and lipoproteins in large arteries; this drives inflammation and fibrosis and ultimately leads to narrowing of the arteries and hypoperfusion of dependent organs and tissues.

Knowledge of the causative factors is crucial to the understanding of disease mechanisms and the development of specific treatment. Based on pathogenetic similarities between PSC and arteriosclerosis, we hypothesize that PSC represents ***"arteriosclerosis of the bile duct" ***initiated by toxic biliary lipids. This hypothesis is based on common molecular, cellular, and morphological features providing the conceptual framework for a deeper understanding of their pathogenesis. This hypothesis should stimulate translational research to facilitate the search for novel treatment strategies for both diseases.

## Background

**Arteriosclerosis**, a disease of the large arteries, represents the single most important contributor to total disease burden in developed countries [1,2]. From a pathomorphological point of view, arteriosclerosis is characterized by smooth muscle cell hyperplasia or hypertrophy and matrix protein accumulation in the vessel intima and media, with lipid deposition leading to thickening and induration of the arterial wall and subsequently to arterial stenosis. Over the past decade, attention has become focused on the critical link between modified lipids and lipid products and the initiation and perpetuation of inflammation in arteriosclerosis [3,4].

**PSC**, a disease of the large bile ducts with a poor median survival of 12 years, is characterised by chronic bile duct inflammation leading to biliary fibrosis and finally cirrhosis. It is often complicated by cholangiocarcinoma (10–15%) [5]. Due to the overall costs to society related to its morbidity, mortality, and need for liver transplantation, PSC in young adults and cholangiopathies in general are increasingly recognized as very important liver diseases [6]. The lack of effective medical treatments for PSC may be deeply rooted in the lack of understanding of the disease mechanisms of PSC.

**Figure 1:**
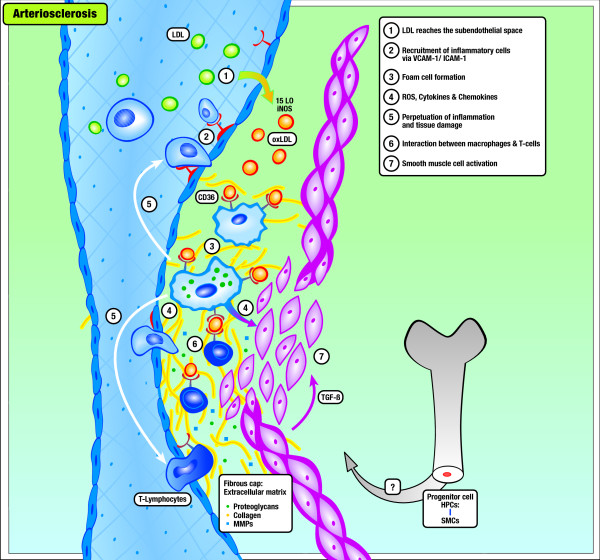
**Model of the Development of Arteriosclerosis**. (1) Low-density lipoprotein (LDL) moves into the subendothelial space and becomes oxidized. (2) Inflammatory cells are recruited into the vessel wall via induced endothelial adhesion molecules (VCAM, ICAM) and (3) take up oxidized LDL and become foam cells. (4) Activation of macrophages leads to the release of inflammatory cytokines, chemokines, inflammatory molecules and reactive oxygen species leading to (5) the perpetuation of inflammation and tissue damage. (6) Antigens presented by macrophages and dendritic cells trigger the activation of cytotoxic T-cells, which produce Th-1 cytokines and heat-shock proteins as well as TGF-β, which activates smooth-muscle cells (7).

**Figure 2:**
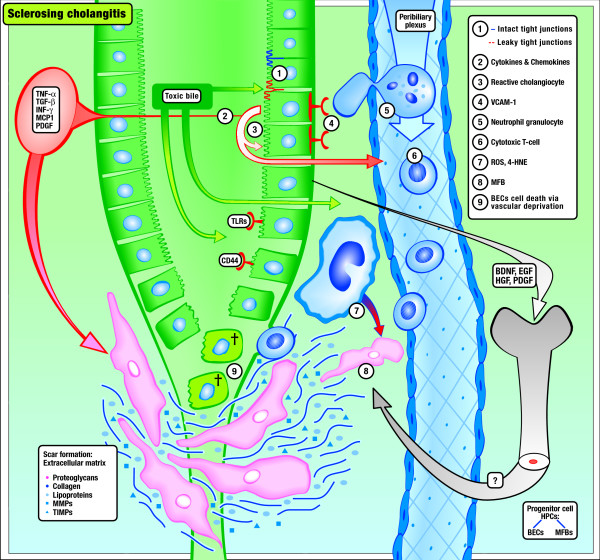
**Model of the Development of Primary Sclerosing Cholangitis**. (1) Toxic bile (e.g. toxic bile acids, oxidized/modified cholesterol and phospholipids) causes (2) the induction of a reactive phenotype of cholangiocytes characterized by *de novo *expression or overexpression of adhesion molecules, inflammatory and profibrogenetic cytokines, and receptors (e.g. TNF-α, TGF-β, toll-like receptors, VCAM, CD44). (3) Proinflammatory cytokines induce leakiness of the bile duct epithelium leading to regurgitation of bile into the portal space, leading to (4) transmigration of (5) neutrophils and (6) lymphocytes and their activation. Bile duct epithelial cell-derived growth factors and cytokines (e.g. TGF-β, PDGF) and reactive molecules released from neutrophils (7) (e.g. ROS, 4-HNE) stimulate extracellular matrix production, accumulation, and proliferation of (8) periductal myofibroblasts, leading to periductal fibrosis and in consequence to vascular deprivation of the bile duct system itself, causing (9) death of bile duct epithelial cells. Bile duct epithelial cell-derived growth factors (e.g. EGF, HGF, PDGF, BDNF) may also activate and recruit bone marrow derived progenitor cells into the portal field probably engaged in ductal reaction and proliferation of periductal MFBs.

Based on similar pathogenetic features and mediators found in both entities (summarized in Table 1 and displayed in Figure 1 and 2, we postulate that PSC shares common disease mechanisms with arteriosclerosis *vice versa *which may become therapeutic targets in the near future in both diseases.

**Table 1:** Factors common to arteriosclerosis and sclerosing cholangitis

**Arteriosclerosis**	**Sclerosing Cholangitis**
**Co-factors** **-Gender**: Age below 60, men develop coronary heart disease twice as frequently as women ***-Infectious agents***: e.g. Chlamydia pneumoniae ***-Systemic inflammation***: elevated CRP levels, associated rheumatoid arthritis	**Co-factors** ***-Gender***: Males are affected twice as often as females ***-Infectious agents***: e.g. increased prevalence of chlamydial antibodies, pos. immunostaining for LPS in bile duct epithelial cells ***-Systemic inflammation***: elevated CRP levels, strongly associated with inflammatory bowel disease

**Abnormal luminal content (blood composition)** -Lipoproteins -Oxidized LDL -Oxidized phospholipids -Cholesterol	**Abnormal bile composition** -Bile acids -Cholesterol (supersaturated bile, oxidized?) -Phospholipids(reduced, oxidized?, metabolites?)

**Activated endothelial cells** ***-Adhesion molecules and receptors***: VCAM-1, ICAM-1, PCAM-1, E-selectin, P-selectin, CD39, CD40, CD44, P2Y, CXCRs, ADAMs, NTFs/NTRKs ***-Cyto- and chemokines***: CXCL, MCP-1, TNF-α, IL-1β, IL-6, IL-8, fractalkine, osteopontin ***-Growth factors***: CTGF, PDGF-β, VEGF, EGF, ET-1, TGF-β ***-Tight junction alterations***: ZO-1, claudin-1, induction of NOS (iNOS)	**Activated bile duct epithelial cells** ***-Adhesion molecules and receptors***: VCAM-1, ICAM-1, E-selectin, CD39L1, CD40, CD44, P2Y, CXCRs, ADAMs, NTFs/NTRKs ***-Cyto- and chemokines***: CXCL, MCP-1, TNF-α, IL-1β, IL-6, IL-8, fractalkine, osteopontin ***-Growth factors***: CTGF, PDGF-β, VEGF, EGF, ET-1, TGF-β ***-Tight junction alterations***: ZO-1, claudin-1, induction of NOS (iNOS)

**Inflammatory cells** ***-Monocytes/macrophage/foam cells***: ROS, IL-6, IL-1, TNF-α, MMPs ***-T cells***: INF-γ, TNF-α, IL-1, IL10	**Inflammatory cells** ***-Neutrophil granulocytes***: ROS, IL-6, IL-1, TNF-α, MMPs ***-T cells***: INF-γ, TNF-α, IL-1, IL10

**Smooth muscle cells** ***-MMPs***: 1, 2, 3, 8, 9, 13,14 ***-ADAMTS***: 1, 13 ***-TIMPs***: 1, 2, 3 ***-Growth factors and chemokines***: HGF, ET-1, TGF-β, ACE2I, RANTES ***-Matrix deposition***: collagen, elastic fibres, glycoproteins, proteoglycanes	**Periductal myofibroblasts** ***-MMPs***: 1, 8, 13 ***-ADAMTS***: 13 ***-TIMPs***: 1, 2, 3, 4 ***-Growth factors and chemokines***: HGF, ET-1, TGF-β, MCP-1, MIP-2, ACE2, RANTES ***-Matrix deposition***: collagen, elastic fibres, glycoproteins, proteoglycanes

**ACE2**, angiotensin converting enzyme II; **ADAM, **a disintegrin and metalloproteinase domain; **ADAMTS**, a disintegrin-like metallopeptidase with thrombospondin type 1 motif; **CD40-**antigen, TNF receptor superfamily; **CD44**, CD44 antigen; **CLDN1**; claudin 1; **CTGF**, connective tissue growth factor; **CXCLs **chemokines, C-X-C motif ligand; **CXCRs**, chemokine C-X-C motif receptors; **CD39/NTPDase-1**, ectonucleoside triphosphate diphosphohydrolase 1;**CD39L1/NTPDase-2**, ectonucleoside triphosphate diphosphohydrolase 2; **EGF**, epidermal growth factor;**ET-1**, endothelin 1; **HGF**; hepatocyte growth factor; **ICAM-1 **intercellular adhesion molecule 1; **INF-γ**, interferon gamma; **IL1-β**, interleukin 1 beta; **IL-6**; interleukin 6; **IL-8**; interleukin 8; **Mcp-1**; monocyte chemoattractant protein-1; **MIP-2**, chemokine (C-X-C motif) ligand 2; **MMPs**, matrix metallopeptidases; **NTFs **neurotrophins; **NTRKs**, neurotrophic tyrosine kinase receptor;**PCAM-1**, polymorph nuclear cell adhesion molecule; **PDGF-β**; platelet-derived growth factor beta polypeptide); **P2Y **purinergic receptor G-protein coupled; **RANTES**, chemokine ligand 5; **TGF-β**, transforming growth factor beta 1; **TIMP1**, tissue metallopeptidase inhibitor 1; **TJP1**, tight junction protein 1; **ZO-1**, zona occludens 1; **TNF-α**, tumor necrosis factor-alpha; **VCAM-1**, vascular cell adhesion molecule 1; **VEGF**, vascular endothelial growth factor.

### Common Pathways in the Pathogenesis of Arteriosclerosis and Primary Sclerosing Cholangitis

Although Virchow first described arteriosclerosis as an inflammatory disorder in 1885 it has taken a long time to become recognised in modern medicine as an active inflammatory disease rather than simply a problem of lipid storage in the vessel wall. In the following we present analogies and potential common pathogenetic mechanisms in the pathobiology of arteriosclerosis and PSC based on recent findings derived from animal models for both diseases [7-10]. Even when it is taken into account that the human body may have a limited repertoire of reactions to noxious and/or infectious stimuli, the similarities between PSC and arteriosclerosis in regard to their pathobiology are stunning and deserve further exploration.

### Exposure to abnormal luminal lipid content: blood versus bile – toxic serum lipids versus cholephiles

All known critical molecular and cellular events underlying the pathogenesis of arteriosclerosis are apparently linked to alterations in lipid homeostasis [3]. As such, the induction of endothelial vascular cell adhesion molecules (Table 1), currently believed to represent the major initiators in arteriosclerosis, is related to the accumulation of oxidized LDL (oxLDL) and phospholipids on the endothelial surface (Figure 1) [3]. As such, oxidized lipoprotein particles (e.g. oxidized phospholipids and short-chain aldehydes arising from lipoprotein oxidation) can induce endothelial VCAM-1 expression via TNF-α- or IL1-β-induced nuclear factor-κB activation [4].

Since the liver is the major organ responsible for the production and degradation of apoB-100-containing lipoproteins, it is most likely that these molecules may also be engaged in the pathogeneses of liver diseases. Moreover, the fact that bile secretion is the most effective means of cholesterol elimination from the human body leads to the suggestion that altered biliary lipid secretion and/or abnormal lipid oxidation leading to oxidative stress may also be critically involved in the initiation and/or perpetuation of PSC. In parallel to arteriosclerosis, enhanced cholangiocellular VCAM-1 expression was recently demonstrated in multidrug resistance gene (*Mdr2/Abcb4*) knockout mice (*Mdr2*^-/-^), a well characterized mouse model that closely mirrors the macroscopic and microscopic pathology of PSC [8,11]. Potentially toxic bile acids synthesized from cholesterol may represent first-line candidates for the induction of cholangitis in *Mdr2*^-/- ^mice [12]. Bile acids are normally packed into mixed micelles together with phospholipids and cholesterol to protect cholangiocytes from potentially toxic bile acids, which may otherwise cause necrosis or apoptosis of cholangiocytes [6]. Absence of phospholipids as a consequence of *Mdr2 *defects not only results in unopposed bile acid toxicity but also in cholesterol supersaturated bile, which could facilitate oxidation. The critical role of **biliary lipotoxicity **in the sense of deranged and consequently toxic bile composition in the development of cholangitis was also underlined by the enhanced cholestatic phenotype in bile acid-fed *Mdr2*^-/- ^mice [13]. Despite the apparent discrepancy between decreased biliary cholesterol concentration in *Mdr2*^-/- ^mice and increased serum cholesterol levels in arteriosclerosis, the common denominator may be abnormal cholesterol oxidation in both conditions. Alternatively, abnormal metabolic products of phosphatidylcholines (e.g. lysoPC) could also be excreted into bile in *Mdr2*^-/- ^mice or reach the cholangiocytes also from the perbiliary plexus or lymph vessels instead from the luminal side. Moreover, bile acids may be able to induce a reactive phenotype of cholangiocytes characterized by the production of several proinflammatory and profibrogenic cytokines and chemokines as well as their corresponding receptors (summarized in Table 1 and shown in Figure 2) [6]. Conversely, some bile acids, their receptors, and synthetic agonists were recently shown to influence the degree of arteriosclerosis in several animal models [14-18]. This fits well with the rapidly progressing insights into the transcriptional regulation by nuclear receptors (e.g. FXR, LXR) of bile acid synthesis/transport on the one hand and lipid metabolism/transport on the other [17,19,20]. Finally, toxic bile acids can activate endothelial cells [21]. Taken together, exposure to abnormal luminal lipid content and composition, ultimately resulting in lipid oxidation, may be critically involved in both conditions.

### Activated endothelial cells versus reactive cholangiocytes – mechanisms of chemoattraction in arteriosclerosis and PSC

Endothelial and bile duct epithelial cells may not only be the victims/targets but also the culprits/effectors that actively participate in inflammatory processes in arteriosclerosis and PSC, respectively. The endothelium represents the first-line contact, where the accumulation of oxLDL induces the expression of adhesion molecules (e.g., VCAM-1) and proinflammatory cytokines/chemokines (e.g., MCP-1, see also Table 1) critically involved in the initiation and perpetuation of arteriosclerosis, making them potential therapeutic targets [4]. Most interestingly, the cytokine/chemokine/receptor armamentarium of reactive endothelial cells and reactive cholangiocytes appears to be largely comparable, as summarized in Table 1[6].

### Altered endothelial versus altered epithelium barrier function – mechanisms of leukocyte penetration and activation

Endothelial binding and penetration of monocytes and T lymphocytes in nascent atheromas by adhesion molecules is pivotal in the development of arteriosclerosis [4]. Moreover, induction of endothelial-derived adhesion molecules reduces barrier function via alterations of the delicately regulated tight junction system, allowing oxidized lipids and marginalized/attached inflammatory cells to migrate into subendothelial regions of the vessel [22]. The MCP-1/CCR2 interaction together with the action of M-CSF represents a critical step for migrated monocytes to differentiate into activated macrophages/foam cells, which themselves are able to produce numerous cytokines, reactive oxygen species (ROS), and growth factors (Table 1). Penetrating T lymphocytes further perpetuate this inflammatory response in a complex orchestrated interplay of cytokines and tissue factors (Table 1, Figure 1).

In analogy to arteriosclerosis, hydrophobic bile acids can induce MCP-1 and TNF-α expression to increase tight junction permeability of cholangiocytes *in vitro *[23,24]. In line with these findings, increased tight junction permeability was also demonstrated in *Mdr2*^-/- ^mice and human cholangiopathies, a mechanism which could permit regurgitation of bile (rich in bile acids, phospholipids, cholesterol, lipoprotein X, CD66a) from the bile duct lumen into the portal tract (Figure 2) [8,25]. Leaking of potentially chemotactic/reactive cholephiles together with enhanced expression and secretion of adhesion molecules may then induce the migration of neutrophils and T lymphocytes from the peribiliary plexus out into the subepithelial space and into the bile duct epithelium, leading to cholangitis (Figure 2). Despite the fact that cytotoxic T cells were shown to induce cholangiocyte apoptosis in humans [26], their role in PSC is still a matter of debate since these cells are virtually absent in precirrhotic PSC stages. The hypothesis becomes questionable that cholangiocytes are the primary target of the immune system in PSC. The role of portal and/or penetrated neutrophils (probably leading to ROS formation and destruction of the basement membrane) in PSC also deserves more detailed studies because these cells may be critically involved in the activation of periductal myofibroblasts by aldehydic end-products of lipid peroxidation.

### Endothelial cell – smooth muscle cell interactions in arteriosclerosis versus bile duct epithelial cell – periductal myofibroblast interactions in PSC

Differentiated monocytes/macrophages and T cells that find their way out through the leaky endothelial barrier exert their pro-atherogenic effects by the secretion of cytokines and growth factors driving smooth muscle cell (SMC) migration and proliferation as well as extracellular matrix (ECM) deposition (Table 1). The development of fibrous plaques results from proliferating SMCs, cholesterol ester and ECM components. This complex interplay is mediated by extensive crosstalk between T-cells and macrophages (CD40/CD40L interactions). Increased homocystein and angiotensin II levels in the vessel wall were also demonstrated to be important mediators of SMC proliferation as well as their activation. In addition, molecules such as osteopontin, CD44 and TGF-β were demonstrated to be critically involved in SMC activation. Reduction of arteriosclerosis in corresponding atherogenic diet-fed knock-out mice [27-29] further underlines the importance of these factors for the development of arteriosclerosis.

A number of these central mediators of arteriosclerosis were also demonstrated to originate from activated cholangiocytes in cholangiopathies [30-32] (Table 1 and Figure 2). Cholangiopathies are frequently accompanied by ductular proliferation/reaction, which is a potential stimulus for biliary fibrosis. The question whether ductular proliferates induce/stimulate proliferation and activation of periductal myofibroblasts and how this might happen (e.g., via stimulation with cytokines such as TGF-β or PDGF, brain-derived peptides such as NGF and BDNF, epithelial-mesenchymal transformation) is critical and still to be resolved.

### Remodelling of the extracellular matrix as self-perpetuating events in arteriosclerosis and PSC

Little is known about the potential importance of the extracellular matrix composition in the pathogenesis of both entities and how modifications (e.g. binding of oxLDL/phospholipids to proteoglycans, cleavage and signalling through proteinases, collagenases, gelatinases, resulting advanced glycosylated endproducts or transglutamination of collagen fibres) could influence the perpetuation of both diseases. In arteriosclerosis, diminished stability of the matrix scaffold is driven by MMPs, collagenases, and the recently identified ADAMTSs (a disintegrin-like metalloprotease with thrombospondin type-1 motifs) [33]. These enzymes could either play a role in the resolution of vessel stenoses or also destabilize plaques [34,35]. In parallel, these enzymes could also be engaged in the chronic persistent wound-healing process in PSC. It is attractive to speculate that chronic activation of MMPs could further increase duct permeability. In addition, persistent activation of ADAMTSs could further increase the inflammatory response through their thrombospondin-1 motive (TSP-1) on TGF-β [36,37] or in the sense of a self-perpetuating process by generating chemotactic/immunogenic ECM products.

### Limitations and caveats using Mdr2^-/- ^mice as a model system for PSC

The exact role of *MDR3 *variants (the human orthologue of rodent *Mdr2*) in the pathogenesis of PSC is still open to question. Two studies (with a total number of 80 PSC patients) found no differences in the number of genetic *MDR3 *variants between healthy individuals and PSC patients [38,39]. The findings of these two genetic studies are consistent with a normal biliary bile acid/PC ratio (indicative of normal MDR3 function) previously reported in 45 PSC patients [40]. However, the number of PSC patients tested so far is still to small to definitely answer the question whether *MDR3 *defects may play a pathogenetic role and reductions in PC excretion may be transient and independent of genetic factors. Moreover, since PSC may represent a complex/polygenetic disease, a major role of *MDR3 *mutations in "unselected" PSC patients would be surprising. Rather, *MDR3 *genetic variations could play a role in selected PSC subgroups (e.g., small duct PSC, young women suffering from PSC, PSC patients with gallstones). Such "unusual" PSC patients could have been missed in previous studies despite the fact that cholelithiasis can be part of its clinical spectrum [41]. Although the role of *MDR3 *variants in the pathogenesis of PSC is still controversial, *Mdr2 *knockout mice may still represent a worthwhile cholangiopathy model. The *Mdr2 *knockout mouse model is the first and so far only non-surgical animal model clearly showing the macroscopic (bile duct strictures and dilatations of the large bile ducts) and microscopic features (onion skin-type like pericholangitis and periductal fibrosis) of sclerosing cholangitis in humans [8,42]. An additional advantage of this model system is the high reproducibility and low variance in the cholestatic phenotype of these animals making them also an ideal model for therapeutic interventional studies [43]. Irrespective of the underlying cause of sclerosing cholangitis, the *Mdr2*^-/- ^model is an appropriate animal model for sclerosing cholangitis since it reliably reproduces the final common pathways of bile duct injury and (peri)biliary fibrosis. However, Mdr2^-/- ^mice do not show some of the (pheno)typical features observed in human PSC such as the association with cholangiocarcinoma and inflammatory bowel disease. finally, the *Mdr2*^-/- ^model may also have general validity as model for cholangiopathies resulting from bile toxicity with a deranged biliary phospholipid/bile salt secretion.

### Differences between PSC and artieriosclerosis

Despite the stunning similarities between PSC and arteriosclerosis in regard to their pathobiology as outlined above, there are also obvious differences between both entities such as the age of onset, different risk factors, and different co-factors (outlined in Table 1) which deserve attention. The exact definition and discrimination of similarities and differences between PSC and artiosclerosis should further help to understand the pathogenesis of both diseases.

### How to test the hypotheses

(i) The toxic bile hypothesis could be tested by studying bile composition with particular emphasis on biliary lipids (e.g. oxidized/modified cholesterol and phospholipids) and other candidate molecules (e.g. CD66a, E-selectin, cyto-, and chemokines), either in bile sampled by endoscopic techniques or assessed via non-invasive modalities such as advanced MR technologies (MR spectroscopy).

(ii) In addition, detailed immunohistochemical studies in PSC livers (either explanted organs or liver biopsy material) using antibodies against adhesion molecules recently identified on cholangiocytes and other well defined mediator molecules in arteriosclerosis such as the receptor for advanced glycosilated end products (RAGE) and oxidized lipids/phospholipids should answer the question of whether these molecules are indeed engaged in the pathogenesis in PSC.

(iii) Ulcerated bile ducts are frequently observed in explanted PSC livers and remind us, at least in some morphological aspects, of plaque rupture in arteriosclerosis. The frequency and importance of duct ulcerations in PSC might be underestimated due to sampling errors inherent to the technique of liver biopsy. Serial sections of explanted PSC livers should help to answer the question of whether concentric bile duct stenoses are the result of a perpetuated wound-healing process of ulcerated/ruptured bile ducts.

(iv) Basic research in PSC should determine the potential role of new inflammatory mediators (e.g. adipokines) known to be critically engaged in the pathogenesis of the metabolic syndrome and consequently arteriosclerosis.

## Conclusion

We postulate a pathogenetic link between PSC and arteriosclerosis. Our model provides a structured and unifying pathogenetic model, based on biological, clinical, and experimental evidence and a network of molecular similarities between both entities. Scientific exchange between researchers/scientists working either in the field of arteriosclerosis or cholangiopathies and cholestatic liver diseases in general should be fruitful and stimulating. Research on PSC may greatly benefit from translating pathogenetic concepts from advanced work in arteriosclerosis to the study of cholestasis. However, this could also work the other way around since it is becoming increasingly apparent that arteriosclerosis may represent a kind of "liver disease of the heart" [44].
